# RELATIONSHIP BETWEEN LONELINESS AND SELF-REPORTED HEALTH AND LIFE SATISFACTION AMONG OLDER ADULTS

**DOI:** 10.1093/geroni/igae098.0823

**Published:** 2024-12-31

**Authors:** Madhyami Deshmukh, Peter Martin

**Affiliations:** Iowa State University, Ames, Iowa, United States; Iowa State University, Ames, Iowa, United States

## Abstract

Loneliness, health, and life satisfaction are important quality of life issues for older adults, but does having children indicate support in quality of life? The aim of this research is to examine the relationship of loneliness on self-reported health, and life satisfaction with number of living children as a moderator. Three recent waves (waves 10, 12, 14) of the Health and Retirement Study (HRS) were used. A total of 14,252 individuals (Mean age = 73) participated in this study. Bivariate correlations and multiple regressions were computed. Results indicated that loneliness at wave 10 negatively correlated with self-reported health at all three waves (all r < = -.20, p <.05) and life satisfaction (all r = < -.22, p <.05). Similar results were obtained at wave 12 and 14, but the associations were stronger for each wave. Loneliness at wave 12 (but not at wave 14) was positively correlated with age. Life satisfaction at waves 10 and 12 was positively associated with age. The multiple regression indicated that loneliness predicted self-reported health (β = -25, p <.001). For life satisfaction, loneliness and number of children predicted life satisfaction (β = -.37 p <.001 and β =.33, p <.001, respectively). Contrary to our hypothesis, the number of children did not moderate the association between loneliness on self-reported health, and life satisfaction. Future implications could help guide better family health planning, better mental health of older adults and opportunities and action plans to informal family caregivers.

